# Live vaccine consisting of attenuated *Salmonella* secreting and delivering *Brucella* ribosomal protein L7/L12 induces humoral and cellular immune responses and protects mice against virulent *Brucella abortus* 544 challenge

**DOI:** 10.1186/s13567-020-0735-y

**Published:** 2020-01-23

**Authors:** Amal Senevirathne, Chamith Hewawaduge, John Hwa Lee

**Affiliations:** College of Veterinary Medicine, Chonbuk National University, Iksan Campus, Iksan, 54596 Republic of Korea

## Abstract

The present study employs the *Brucella abortus* L7/L12 antigen in a *Salmonella* secretion platform and investigates its ability to induce protective immune responses against wild type challenge in BALB/c mice. The highly conserved L7/L12 open reading frame was PCR amplified from *B. abortus* and cloned into a prokaryotic expression vector, pJHL65, directly under the beta-lactamase secretory signal. The plasmid constructs pJHL65::L7/L12 was then transformed into an attenuated *Salmonella* Typhimurium strain, JOL1800 (*∆lon*, *∆cpxR*, *∆asd*, and *∆rfaL*), and protein secretion was verified by Western blot. Three mice groups were inoculated with either phosphate-buffered saline (PBS), vector-only control, or the vaccine strain secreting L7/L12 antigen. Assessment of humoral and cell-mediated immune responses revealed successful elicitation of *Brucella* antigen-specific Th1 and Th2 immune responses that were significantly higher than PBS and vector control groups. The immune responses were confirmed by splenocyte proliferation assay, flow cytometry analysis for CD4+ and CD8+ markers, and RT-PCR based cytokine profiling upon restimulation with L7/L12 purified antigen. Results indicate that immunization with *Salmonella* secreting L7/L12 antigen demonstrated significant enhancement of cell-mediated immune (CMI) responses in immunized mice. The overall effectiveness of the immunization was evaluated by challenging with virulent *B. abortus* that revealed significant reduction in *Brucella* colonization in spleen and liver tissues in *Salmonella* L7/L12 immunized mice. Delivery of *Brucella* protective antigen L7/L12 using the *Salmonella* secretion system can effectively accomplish immunogenic advantages of both *Salmonella* and L7/L12 to derive robust CMI responses and induce humoral immunity to protect against *Brucella* infection in the mouse model.

## Introduction

*Brucella abortus* is a zoonotic bacterial pathogen within the genus *Brucella*. It induces abortions, placental retention, and infertility in ruminants and undulant fever in humans with occasional chronic infections. As an intracellular pathogen, it proliferates in both phagocytic [1] and non-phagocytic cells [2] in the infected host. Host resistance to *B. abortus* mainly depends on interferon-gamma (IFN-γ) involved cell-mediated immune responses [3]. Up regulation of IFN-γ cytokine is essential to induce the anti-*Brucella* activity of macrophages that helps to clear *Brucella* from the infected host [4].

Among antigens that efficiently induce anti-*Brucella* cell-mediated immune (CMI) responses, *Brucella* ribosomal protein L7/L12 has been recognized as a plausible candidate in recent research studies. These experiments have shown that L7/L12 can efficiently induce protective immune responses in the form of subunit or DNA vaccines [5, 6]. Apart from CMI responses, antibody-mediated humoral responses also play a vital role during *B. abortus* infection. Elicitation of mucosal immune responses is effective during the early phase of infection on mucosal surfaces, where the complement killing system can reduce the proportion of *Brucella* entering the intracellular environment [7]. Therefore, an effective prevention strategy for *B. abortus* should ideally harness both CMI responses and humoral immune responses in the immunized host [8]. Utilizing an attenuated systemic pathogen such as *Salmonella* for the delivery of protective antigens can capitalize on both CMI and humoral responses considering their mechanism in host immune stimulation. Based on the above fact, here we utilize *Salmonella* to deliver *Brucella* antigen (L7/L12). As an intracellular pathogen, *Salmonella* can invade into host macrophages and lymphoid organs such as the spleen and liver [9]. Thus, *Salmonella* can effectively deliver heterologous antigens directly into macrophages, other cells, and tissues where antigen-presenting cells can process and recognize *Brucella* antigens for adaptive immune responses and fight against infections. Further, *Salmonella* can overcome mucosal barriers and actively penetrate the host tissue environment that is essential to maximize antigen presentation [10]. In addition, sufficient attenuation can also be incorporated into the *Salmonella* genome with relative ease due to its genome that has been thoroughly investigated with most virulence-related genes being identified.

Despite scientific advancement in DNA vaccine technology during recent years, plasmid delivery in in vivo conditions remains a significant challenge. Intracellular delivery, entry into the cell nucleus, and expression of the intended protein is a relatively inefficient process. To overcome such inefficacy in DNA vaccines, high quantities of plasmid DNA usually have to be utilized with multiple booster applications [11]. Such inconveniences may be avoided by using an efficient *Salmonella* mediated secretion system that does not require induction or multiple immunizations. In our previous study, an attenuated *Salmonella* Typhimurium strain, JOL1800, was constructed with specific deletions in the *lon*, *cpxR*, and *rfaL* genes. The resulting strain has impaired intracellular survival due to loss of *lon* and *cpxR* virulence-related genes [12] and has a truncated lipopolysaccharide structure due to a *rfaL* gene mutation. The resulting JOL1800 DIVA (Differentiating infected from vaccinated animals) is ideal for vaccine studies. Besides, this strain is aspartate dehydrogenase auxotrophic (*Δasd*) and can stably harbor plasmid constructs complemented with *asd* gene [13].

The aim of the present study is to design a safe *Brucella* vaccine that is applicable in both human and animal models for efficient elicitation of humoral and cell-mediated immune responses utilizing a *Salmonella* secretion and delivery system. The present *Salmonella* strain, JOL1800, is an *asd* auxotrophic mutant that can stably carry an *asd* complemented plasmid pJHL65, which contains fusion of *B. abortus* ribosomal protein L7/L12 with the beta-lactamase (*bla*) secretory signal sequence. Attenuated *Salmonella* can secrete heterologous *Brucella* antigens into the host by crossing the periplasmic barrier acting as an intracellular pathogen. Due to impaired virulence, attenuated *Salmonella* does not cause systemic infection and only provides transient immune stimulation in the host. We have observed robust elicitation of both humoral and cellular immune responses in immunized mice with significant protection against virulent challenges. We have observed *Salmonella* alone carrying the empty plasmid vector provide considerable protection against the virulent *Brucella* challenge that may be attributed to *Salmonella* induced immune stimulation. The results indicate that *Salmonella* is an excellent antigen delivery system for specific induction of anti-*Brucella* immunity that takes advantage of both *Salmonella* and L7/L12 immunogenic capabilities. This provides an easy and effective strategy for mass production of an anti-*Brucella* vaccine that is applicable to both humans and animals. In the present study, we demonstrated that a *Salmonella* system could be effectively utilized for delivery of *Brucella* ribosomal protein L7/L12. This elicits robust anti-*Brucella* protective immunity by achieving the immunogenic advantages of *Salmonella* and L7/L12 as a protective antigen to be used in the battle against *Brucella* infection.

## Materials and methods

### Bacterial strains and plasmids

The bacterial strains, plasmids, and primers used in this experiment are listed in Table 1. Attenuated *Salmonella* and *Escherichia coli* strains were routinely grown at 37 °C in Luria–Bertani (LB) broth or agar with or without appropriate antibiotics unless otherwise indicated. *B. abortus* 544 strain was grown in *Brucella* medium (BD, Sparks, USA) at 37 °C in a 5% CO_2_ atmosphere without antibiotics. Appropriate biosafety measures were enforced while handling *B. abortus*, a biosafety level 3 microorganism.

**Table 1:** **Bacterial strains, plasmids, and primers**

Strain/Plasmid	Description	Reference
*S*. Typhimurium		
JOL1800	*∆lon, ∆cpxR, ∆asd* and *∆wpaB* mutant of *S.* Typhimurium	Lab stock
JOL2273	JOL1800 containing pJHL65:: L7/L12 and expressing l7/L12	Lab stock
JOL2080	JOL1800 carrying the empty vector	Lab stock
*E. coli*		
BL21(DE3)pLysS	F^−^, *omp*T, *hsd*S_B_ (r_B_^−^, m_B_^−^), *dcm, gal*, λ (DE3), pLysS, Cm^r^	Progma, USA
JOL1921	BL21(DE3)pLysS harboring pET28a + :: L7/L12	Lab stock
Plasmid		
pET28a(+)	IPTG-inducible expression vector; Kanamycin resistant	Novagen, USA
pET28a + :: L7/L12	pET28a + derivative containing L7/L12	Lab stock
pJHL65	*asd*^+^ vector, pBR ori, β-lactamase signal sequence-based periplasmic secretion plasmid, 6xHis, high copy number	Lab stock
pJHL65:: L7/L12	pJHL65 harboring L7/L12	Lab stock
Primer		
L7/L12 EcoRI F	5′-AGAGGAATTCATGGCTGATCTCGCAAAGAT-3′	This study
L7/L12 HindIII R	5′-AGAGAAGCTTTTACTTGAGTTCAACCTTGG-3′	This study

### Construction of attenuated *Salmonella* mutant delivering *Brucella* antigen

The construction of the *asd* complemented pJHL65 plasmid that constitutively expresses heterologous antigens was described in a previous report [7]. Briefly, *Brucella* ribosomal protein L7/L12 gene was PCR amplified from the *B. abortus* genome and cloned in-frame immediately downstream to the beta-lactamase (*bla*) secretory signal. Thereby, proteins can be externalized using a Type II secretory system. The novel plasmid construct was denoted as pJHL65:: *L7/L12*. The recombinant plasmid was then transformed into an *asd* mutant auxotrophic *Salmonella* mutant strain JOL1800 (*∆lon*, *∆cpxR*, *∆asd*, and *∆rfaL*). The resultant strain was designated as JOL2273. The presence of the respective gene in the attenuated *Salmonella* strain was confirmed by PCR. Secretion of *Brucella* ribosomal protein L7/L12 by the attenuated *Salmonella* strain was confirmed by Western blot analysis utilizing HRP labeled anti-his tag antibody. To generate purified protein, the L7/L12 coding sequence was cloned into a pET28a+ vector (Novagen, Madison, WI, USA). The recombinant plasmid was then transformed into *E. coli* BL21 DE strain (Novagen) for overexpression and purification. The expressed protein was purified using Ni_NTA (Qiagen, Valencia, CA, USA) affinity column chromatography (Poly-prep, Bio-Rad, Hercules, CA, USA) according to the manufacturer’s instructions. The concentration of purified protein was then quantified by Bradford assay and stored at −20 °C for further use.

### Immunization and challenge

All animal experiments were approved by the Chonbuk National University Animal Ethics Committee (CBNU-2018-00264) in accordance with the guidelines of the Korean Council on Animal Care and Korean Animal Protection Law, 2007; Article 13. All mice used in the study were housed and maintained humanely with the ad libitum supply of antibiotic-free food and water. Five-week old specific pathogen-free female BALB/c mice (*n* = 10) were randomly divided into three groups: PBS control group, vector-only group, or L7/L12 treatment group. Each mouse group was intra-muscularly immunized with phosphate-buffered saline (PBS), *Salmonella* JOL1800 bearing only the vector, or treatment strain harboring pJHL65::L7/L12 at 1 × 10^7^ CFU/mice in 100 µL of PBS. On the 14th day post-immunization, blood samples for serum preparation [14, 15] and vaginal washes were collected [7] for IgG and sIgA and subtype IgG and IgG2a antibody assessment (*n* = 10). In addition, on the 14th day, five mice from each group were sacrificed for splenocyte collection and used for splenocyte proliferation assay, flow cytometry analysis, and RT-PCR assays. Twenty-one days post-immunization, the remaining mice (*n* = 5) from each group were intraperitoneally challenged with virulent *B. abortus* 544 strain at 2 × 10^5^ CFU/mouse. After 14 days of challenge, all mice were euthanized. The total *Brucella* load in whole spleen and liver organs were enumerated by counting colony-forming units using macerated tissue samples as described before [7].

### Antigen-specific IgG and secretory IgA responses

Indirect ELISA (enzyme-linked immunosorbent assay) was used to assess L7/L12 specific IgG and secretory IgA (sIgA) responses in sera and vaginal wash samples, respectively, according to a previous report [16] with minor modifications. Briefly, purified L7/L12 protein (300 ng/well) was coated on the ELISA plate overnight at 4 °C in a coating buffer. Plates were washed and blocked with 5% skim milk. Serum samples (1:50) and vaginal washes (without dilution) were incubated for 1 h at 37 °C. Unbound antigen was washed, and plates were incubated with anti-his tag HRP (1:3000). Color development was achieved by adding o-phenylenediamine dihydrochloride (OPD) substrate and was measured at 492 nm wavelength. Further, serum collected at 3rd week post-immunization was assessed in ELISA against L7/L12 purified protein, *Brucella* whole cell lysate and *Salmonella* whole cell lysate following the same procedure.

### Antigen-specific splenocyte proliferation assay

Antigen-specific cell-mediated immune responses (CMI) were evaluated in a splenocyte proliferation assay. Splenocytes from mice (*n* = 5) in each PBS control, vector alone, and *Salmonella* L7/L12 groups were harvested at 2 and 3 weeks of post-immunization. Cell number was adjusted to 1 × 10^5^ cells/well in 96-well plates and seeded in replicates using RPMI medium supplemented with 10% FBS. Next, cells were stimulated with specific purified L7/L12 protein 10 μg/mL or RPMI media alone. Three days later, cell proliferative response was evaluated by MTT [3-(4,5Dimethylthiazol-2-yl)-2,5-diphenyltetrazolium bromide] based assay as described in an earlier report [17]. The stimulation index was calculated compared to the responses received in PBS control group. To further confirm the CMI response, the same experiment was repeated at 3rd week post-immunization and stimulation either with purified *Brucella* L7/L12 protein or *B. abortus* whole cell lysate (10^7^ CFU/mL). Splenocytes collected at 3 weeks post-immunization were tested against L7/L12 purified protein and *Brucella* whole cell lysate for comparison.

### Cytokine responses

Splenocytes from immunized mice were aseptically harvested using five mice per group. Splenocytes (1 × 10^6^ cells/mL) were stimulated with 200 ng of the respective purified L7/L12 antigen protein for 24 h. Total RNA was isolated by RNeasy Mini Kit (Qiagen, Hilden, Germany) according to the manufacturer’s instructions. Complementary DNA was prepared from 1 μg of RNA using ReverTra Ace qPCR RT Master Mix (TOYOBO, Osaka, Japan) according to the manufacturer’s instructions and stored at −20 °C until use. Real-time PCR assay (qRT-PCR) for gene expression studies was performed with ABI applied biosystems using Power SYBR Green PCR Master Mix (#4367659, Applied Biosystems, USA) as previously described [18]. The relative amounts of IFN-γ, IL-4, and IL-17 were determined by 2^−ΔΔCT^ method [19].

### Flow cytometry

Flow cytometric analysis of CD4+ and CD8+ T cell populations were carried out on mice splenocytes (*n* = 5) harvested on the 14th-day post-primary immunization, as previously described [7]. Briefly, 2 × 10^5^ viable cells were stimulated with *Brucella* whole cell lysate antigen (10 μg/mL) or RPMI media alone for 24 h at 37 °C in 5% CO_2_ atmosphere. Cells were then harvested and stained with PE-labeled anti-CD3e, PerCPVio700-labeled anti-CD4, and FITC-labeled anti-CD8a monoclonal antibodies, as previously described [20]. The CD3+ T cell population was gated to analyze CD3+ CD4+ and CD3+ CD8+ subpopulations in both vaccinated and control mice groups using a MACSQuant^®^ analyzer (Miltenyi Biotec, Bergisch Gladbach, Germany).

### Statistical analysis

All data were analyzed using the GraphPad Prism 6.00 program (San Diego, CA, USA). One way analysis of variance (ANOVA) with Tukey’s multiple comparison test was conducted to determine statistical differences among vaccinated and control groups. *P* values < 0.05 were considered statistically significant.

## Results

### Confirmation of *Salmonella* secreted *Brucella* antigen

The gene of *B. abortus* ribosomal protein L7/L12 was amplified by PCR using *B. abortus* 544 strain and cloned into the pJHL65-*asd* complemented plasmid (Additional file 1A). The insertion of the L7/L12 gene cassette (0.374 kb) was confirmed by PCR amplification and double digestion of host plasmid with EcoRI and HindIII restriction enzymes. The presence of the plasmid in *Salmonella* transformants was confirmed by colony PCR method. The pJHL65 plasmid provides *bla* secretion signal upstream to the L7/L12 gene cassette. The efficacy of protein secretion was confirmed by Western blot analysis of the culture supernatant, which yielded the target protein antigen with approximately 12.5 kDa (Additional file 1B).

### *Brucella* antigen-specific systemic IgG and secretory IgA antibody response

**Figure 1:**
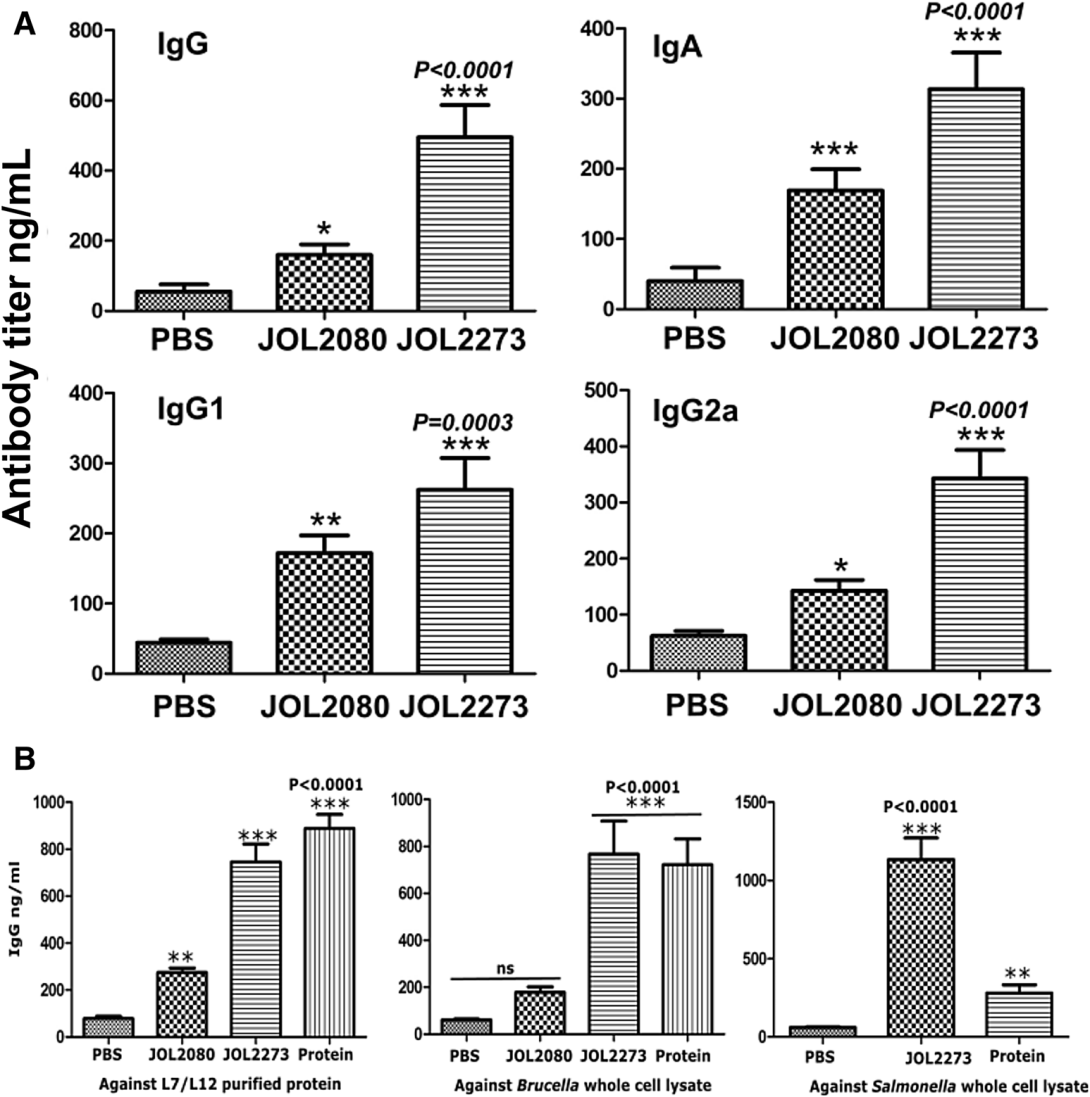
**Humoral immune responses.** Five-week old female BALB/c mice were immunized with PBS, vector-only strain, and *Salmonella* secreting *Brucella* L7/L12. Humoral responses in serum and vaginal washes were measured on 14th day post-immunization. **A** Serum IgG response, IgA response in vaginal washes, and serum IgG1 and IgG2a were demonstrated. *Indicates significant difference compared to PBS control (*P* < 0.05). Probability values were depicted. **B** The IgG responses at 21st day post-immunization were measured against purified L7/L12 antigen, *Brucella* whole cell lysate and *Salmonella* whole cell lysate were conducted. ***Indicates the significant difference against the PBS control (*P* < 0.05).

The degree of host response towards foreign antigen L7/L12 was assessed by measuring the antigen-specific antibody response in blood serum and mucosal secretions. Efficient elicitation of both IgG and sIgA (Figure 1A) responses indicates the effectiveness of *Salmonella* delivered antigen presentation. Antibody concentrations on the 14th-day post-immunization demonstrate a significant high IgG and sIgA response in the L7/L12 treated mice group compared to the PBS and vector control groups. The specific elicitation of IgG1 and IgG2a antibody subtypes indicates engagement of both Th1 and Th2 type immune response upon *Salmonella* immunization. However, concerning IgA response, and IgG1 and IgG2a antibody subtype response, a significantly high antibody elicitation is evident due to *Salmonella* vector alone. This suggests that approximately 50% of IgA response, 55% IgG1, and 45% IgG2a response elicited by the *Salmonella* secreting L7/L12 is attributed to *Salmonella* itself. Even though the vector alone elicits L7/L12 specific humoral responses, it is clear after statistical comparison that the *Salmonella* L7/L12 strain still generates significantly higher humoral response even after the subtraction of the vector alone responses. Further, to dissect whether L7/L12 specific antibodies can cross react with *Salmonella*, a repeat experiment was carried out by inoculating mouse with purified protein along with PBS, *Salmonella* L7/L12 and *Salmonella* alone controls. Serum was collected after 3 weeks post-immunization tested against L7/L12 purified protein, *Brucella* whole cell lysate and *Salmonella* whole cell lysate (Figure 1B). Herein, the *Salmonella* vector alone response showed a reduced signal against *Brucella* whole cell lysate probably due to competing antigens and impurities present in the lysate. Further, this result confirms that *Brucella abortus* L7/L12 demonstrates a positive reaction against *Salmonella* whole cell lysate suggesting the fact that there can be a cross reaction between L7/L12 antibodies derived against *Salmonella* native L7/L12 antigen and *Salmonella* secreted *Brucella* L7/L12 antigen. It also can be hypothesized, that the antibodies developed against *Brucella* L7/L12 can result in a cross reaction with native *Salmonella* L7/L12 antigen due to higher amino acid sequence identity and homology present between the two species (Additional file 4).

### Cell-mediated immune responses

**Figure 2:**
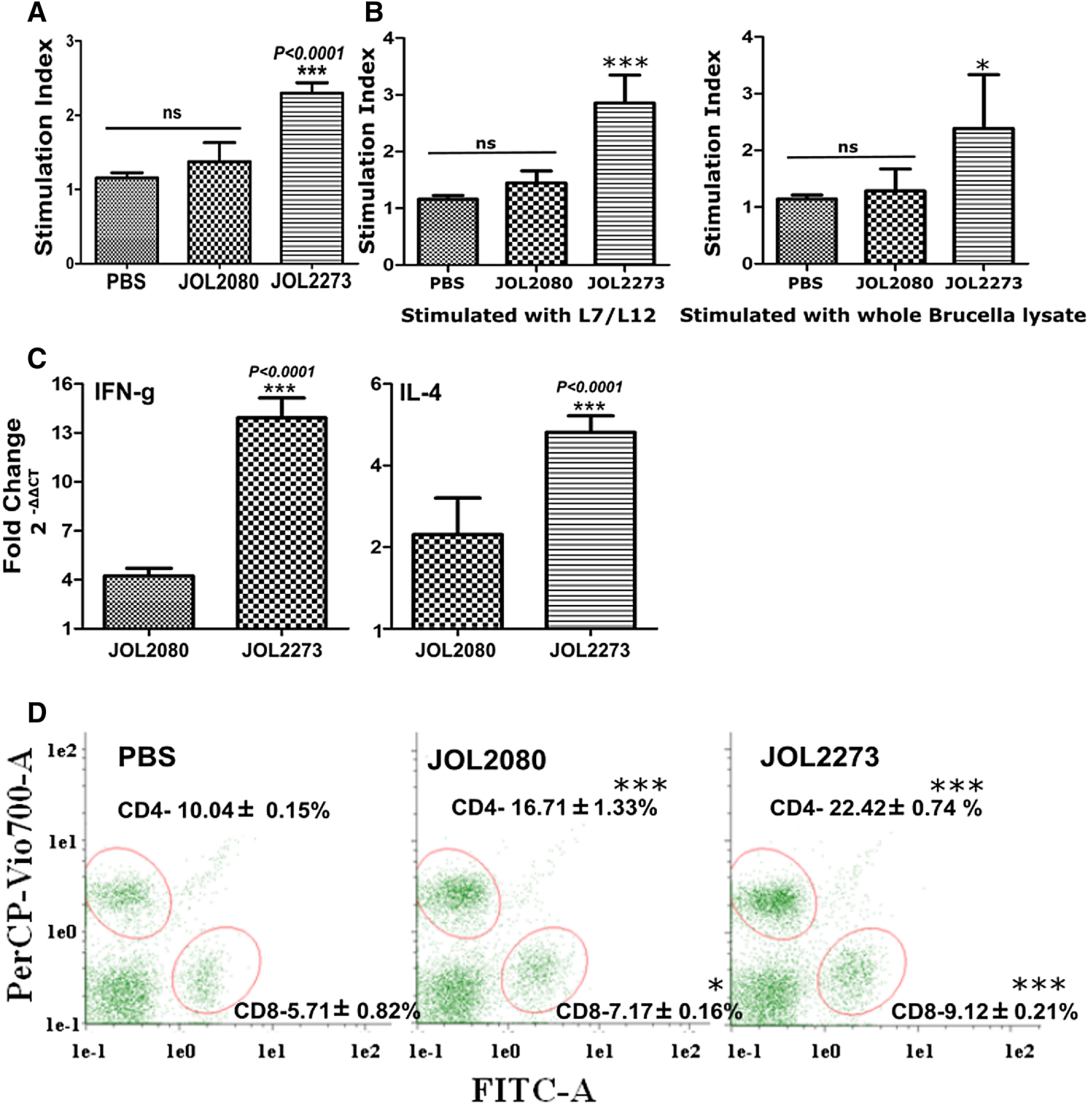
**Cell mediated immune responses. A** Stimulation index. Mice were immunized with PBS, vector-only strain, and *Salmonella* secreting *Brucella* L7/L12. Two weeks post-immunization, 5 mice from each group were sacrificed, and splenocytes were aseptically collected and restimulated with immunizing antigen. Cell proliferative response was measured by MTT based assay after 3 days of incubation. ***Indicates significant difference compared to PBS control; ns: non-significant difference. Significance was determined at *P* < 0.05. Exact probability is demonstrated on graph. **B** Stimulation index at 3 weeks. The stimulation index was determined at three weeks of immunization against purified L7/L12 antigen and whole *Brucella* cell lysate. ***Indicates the significant difference against the PBS control, ns: non-significant difference. **C** Cytokine response. Mice were immunized with PBS, vector-only strain, and *Salmonella* secreting *Brucella* L7/L12. Two weeks post-immunization, 5 mice from each group were sacrificed, and splenocytes were aseptically collected and restimulated with immunizing antigen. After 1 day of incubation, interferon-gamma and IL-4 response were evaluated at mRNA level by quantitative RT-PCR. Fold change of expression is demonstrated. ***Indicates significant difference against PBS control group. Level of significance was determined at *P* < 0.05. Exact probability value is demonstrated on the graph. **D** Flow cytometry. Mice were immunized with PBS, vector-only strain, and *Salmonella* secreting *Brucella* L7/L12. Two weeks post-immunization, 5 mice from each group were sacrificed, and splenocytes were aseptically collected and restimulated with immunizing antigen. After 1 day of incubation, cells were labeled with CD3+. CD4+ and CD8+ specific anti mouse antibodies CD3+: PE, CD4+: PerCP Vio700, CD8+: FITC (Miltenyi Biotec, Gladbach, Germany) and analyzed in Macsquant flow cytometer instrument (Miltenyi Biotec, Gladbach, Germany). Each population was gated and quantified. ***Indicates significant difference against PBS control group.

The splenocyte proliferation assay is an indicator of CMI responses. The *Brucella* L7/L12 antigen is known to induce CMI responses. Thus, the retained biological activity of *Salmonella* secreted *Brucella* L7/L12 can be assessed by a MTT based splenocyte proliferation test. On the 14th day post-immunization, the L7/L12 treated group had significantly high cell proliferation response after re-stimulation of cells with purified L7/L12 protein compared to the PBS and vector control groups (Figure 2A). When the splenocytes were collected at 3 weeks post-immunization and stimulated by L7/L12 purified protein or *Brucella* whole cell lysate, proliferation test results demonstrated a higher specificity towards L7/L12 antigen and reactivity against both purified L7/L12 protein and *Brucella* whole cell lysate at 3rd week post-immunization. This result indicates that *Salmonella* secreted L7/L12 elicits cell mediated immune responses that react when encounter immunizing antigen. The engagement of both Th1 and Th2 immune responses could be assessed by analyzing cytokine profiles. Herein, representative cytokines IFN-γ and IL-4 expression levels show significant increase compared to the PBS control. Here too, vector alone responses also resulted in a significant difference from the PBS group. These results suggest that immunization with *Salmonella* secreting L7/L12 elicits a skewed response towards Th1 immune response over Th2 immune response, signifying an essential IFN- γ mediated cellular responses (Figure 2C). Furthermore, flow cytometry analysis of splenic T-cell populations after 14 days of immunization showed a significant increase in both CD4+ and CD8+ expressing cell populations. It suggests that *Salmonella* secreting *Brucella* L7/L12 antigen activates and increases antigen-specific T-cell responses in the host. The present results also argue that *Salmonella* alone elicits a considerable immune response against L7/L12 antigen stimulation.

### Protection efficacy against virulent *Brucella* challenge

**Figure 3:**
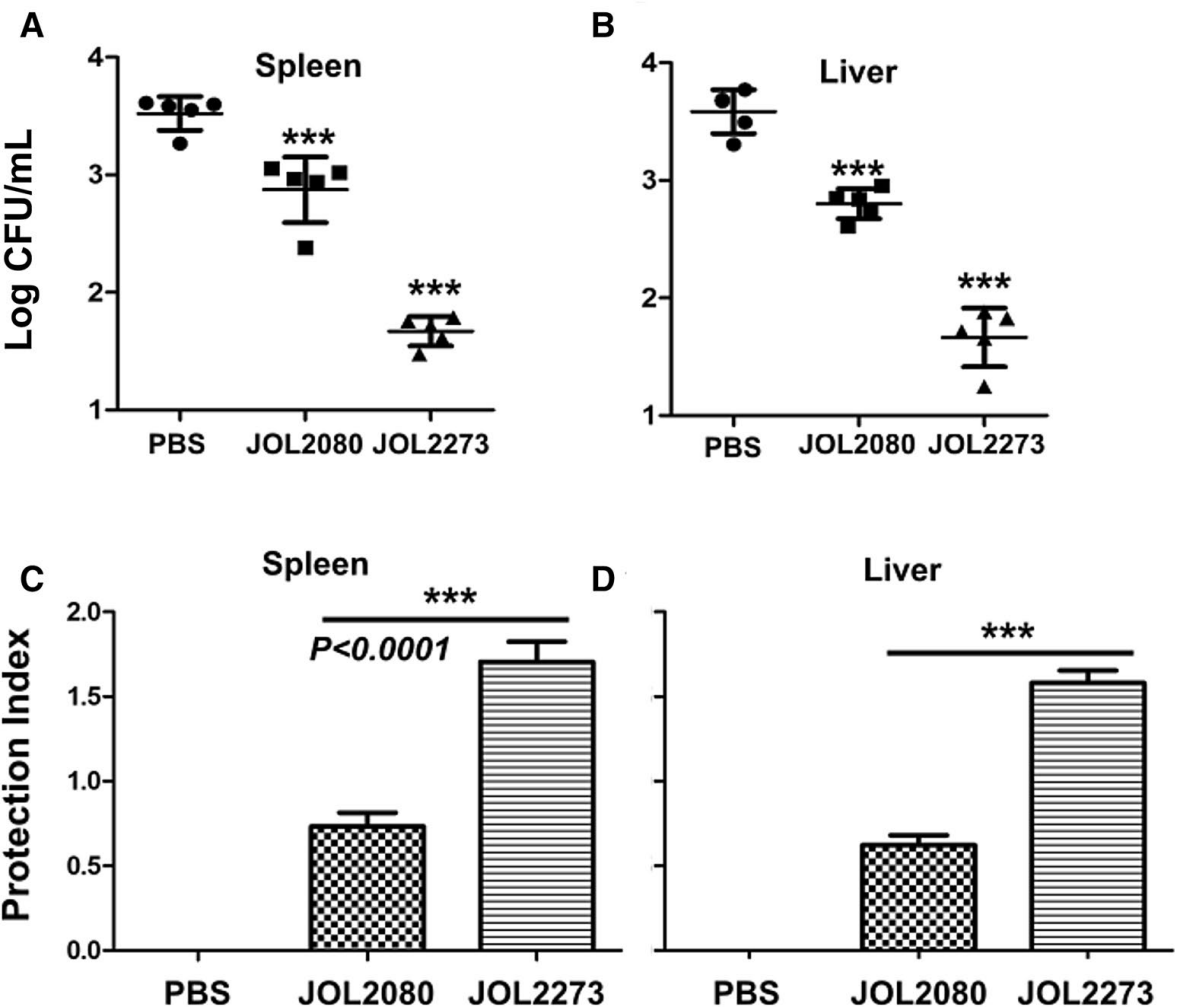
**Protection index.** Protective efficacy was determined after 2 weeks of challenge conducted on 21st day post-immunization. *Brucella* colony count was taken in spleen (**A**) and liver (**B**) tissues in each group (*n* = 5). Colony counts were converted into protective index using the statistical formula (y = log(x/log x), PI = y_pbs_ − y_test_) and presented with standard deviation. ***Indicates the significant difference against PBS control. *P* < 0.05 was considered significantly different.

To investigate the protective efficacy of *Salmonella* delivered L7/L12 antigen against virulent *B. abortus* challenge, all immunized and control mice were challenged with *B. abortus* 544 strain. Two weeks after the challenge, *Brucella* bacterial burden was quantified by plate method with serially diluted tissue samples (Figure 3). Our results indicate approximately 2 log reduction in bacterial numbers in both spleen and liver samples of the L7/L12 treatment group compared to the PBS and vector-only control groups (Figures 3A and B). Protective index was calculated by using the statistical formula (y = log(x/log x), PI = y_pbs_ − y_test_) (Figures 3C and D). In the protection index too, *Salmonella* vector control resulted in a significantly high level of protection compared to the PBS control.

## Discussion

The present study uses the highly immunogenic *Brucella* ribosomal protein L7/L12 in a *Salmonella* secretion platform as a live attenuated vaccine for brucellosis. The protective efficacy against virulent *Brucella* challenge was assessed in a BALB/c mice model. The combined elicitation of humoral and cell-mediated immune response clearly suggests that the combination of L7/L12 antigen with a *Salmonella* delivery system can increase the immune stimulation effect of both entities, *Salmonella* and L7/L12. The selected antigen L7/L12 consists of several dominant antigenic epitopes that are surface exposed (Additional file 2). Thus, efficient antigen processing and presentation are expected. Another significant advantage of L7/L12 ribosomal protein is its high sequence conservation, with more than 99% of its amino acid sequence identified in members of the *Brucella* genus (Additional file 3). Hence, cross-protection is also anticipated. In the present vaccine design, L7/L12 is incorporated into the *Salmonella* type II secretion system [21] via fusion with *bla* signal sequence and under the constitutive Ptrc expression promoter. This results in continuous antigen secretion into the surroundings (Additional file 1B). This may enhance the chances of antigen presentation by *Salmonella* secreting L7/L12 antigen as the *Salmonella* can penetrate the host tissues including the lymphoid organs such as spleen and liver [22, 23].

Despite recent research advancements in DNA vaccine technology, the efficacy of DNA delivery remains poor and insufficient for single-dose immunization [24]. This is true for both living and non-living DNA carriers during immunization procedures. Immunizations in the form of purified proteins have their limitations, such as high processing cost, requirement for multiple booster applications, and potential for degradation [25]. Such practical inefficacies were minimized in the present *Salmonella* mediated immunization strategy because the antigen is readily secreted by *Salmonella* and is available for immune stimulation (Additional file 1B). IFN-γ mediated cellular immune responses are considered essential factors for efficient elimination of intracellular *Brucella* [5, 26]. This is the main objective expected from *Brucella* ribosomal protein L7/L12 as it can induce essential CMI responses for intracellular elimination of *Brucella* [7, 11]. In the present study, immunization with *Salmonella* secreting *Brucella* ribosomal protein in a single dose clearly induced both IgG and IgA (Figure 2) humoral antibody responses [20]. These responses play a vital role in host protection during the early phase of *Brucella* infection on mucosal surfaces [27]. On mucosal tissues, complement-mediated killing effectively reduces internalization of *Brucella* pathogen into epithelial cells. Once *Brucella* becomes an intracellular pathogen, the IFN-γ cytokine-mediated Th1 immune response is crucial [28]. In the present investigation, immunization with *Salmonella* secreting *Brucella* L7/L12 protein induced both CMI (Figures 2A and B) and IFN-γ (Figure 2C) responses significantly more than in PBS and vector control mice. An increase in the frequency of CD4+ and CD8+ T-cell populations also provides evidence for both Th1 and Th2 immune responses. Particularly, stimulation of CD8+ markers on T-cells can be attributed to L7/L12 induced Th1 immunity, as they were significantly higher in L7/L12 immunized mice than in the vector control group. The challenge conducted with virulent *B. abortus* 544 strains showed clear reduction in *Brucella* populations in spleen and liver tissues in the *Salmonella* vaccine immunized group compared to the vector and PBS control groups, resulting in a significantly higher protection index (Figures 3A and D) and demonstrating the overall effectiveness of the immunization among test groups.

Considering the antigen specific immune responses in the form of humoral, CMI, and cytokine induction, *Salmonella* vector alone has delivered a considerably high expression levels. We hypothesize this observation may occur particularly due to amino acid sequence and epitope conservation of L7/L12 antigen between *Brucella* and *Salmonella* species (Additional file 4). These two species not only share highly similar antigenic epitopes but also demonstrate over 57% amino acid sequence identity (95% query cover × 60.50% percent identity) suggesting the possibility for antibody cross reactions generated in mice against the native *Salmonella* L7/L12 and *Salmonella* secreted *Brucella* L7/L12 antigen. However, the reduction in bacterial colonization in spleen and liver tissues by *Salmonella* vector control may be partially attributed to *Salmonella* induced non-specific immune stimulation signified by increased IFN-γ response.

Safety considerations play a crucial role before live vaccine strains can reach clinical studies. The present JOL1800 strain did not induce any mortality in immunized mice due to attenuation caused by the deletion of *lon*, *cpxR*, and *rfaL* genes [13, 29]. The safety of the strain has been extensively proven in our previous studies conducted in mice models. Adding further advantage to the vaccine strain, deletion of the *rfaL* gene confers DIVA capability [13] due to removal of a major immunodominant component from the O-antigen. Therefore, the vaccine may be applied with minimum effects from pre-existing lipopolysaccharides (LPS specific) anti-*Salmonella* immunity of the immunizing host [13].

In the present study, we demonstrated that mice immunized with *Salmonella* secreting *Brucella* ribosomal protein L7/L12 could harness both humoral and CMI responses essential for the effective elimination of intracellular pathogen *Brucella.* Here, a single dose of immunization provides a practically convenient vaccination strategy suitable for field conditions. Further research can investigate *Salmonella* secreting L7/L12 fusion proteins with other immunodominant *Brucella* antigens, such as SodC and Omp19 [9], which may further augment L7/L12 mediated protective immunity.

## Supplementary information

**Additional file 1.** **Graphic representation of plasmid construct and confirmation of protein secretion.** (A) Major elements of plasmid pJHL65:: L7/L12 are depicted. Figure is not drawn with actual proportions. (B) Western blot confirmation of protein secretion. Expected size of L7/L12 protein is 12.5 kDa.

**Additional file 2.** **Epitope analysis of L7/L12 is demonstrated.** (A) Graphic representation of predicted epitopes is presented in yellow. (B) Distribution of predicted epitopes on three-dimensional model is demonstrated. (C) Amino acid residues resembled in each epitope are demonstrated in red. Altogether, 5 immunodominant epitopes were predicted.

**Additional file 3.** **High degree of L7/L12 sequence conservation is demonstrated within** ***Brucella*** **taxid**. Only variable residues were demonstrated in white boxes. Phylogenetic tree constructed on maximum likelihood method also demonstrates extremely high level of sequence identity. Branch length resembles the number of amino acid residue substitutions in each species. Figures were generated by Phylogeny.fr program and Unipro UGENE.

**Additional file 4.** **Sequence homology.** (A) The homology of B cell epitopes were compared between *Brucella* and *Salmonella* L7/L12 antigen. (B) The sequence identity was determined between *Brucella* and *Salmonella* L7/L12 antigen using Muscle algorithm in Ugene software. The squares depict the predicted B-cell epitopes on *Brucella* L7/L12 antigen.
