# Supplementary figures and images for: Live vaccine consisting of attenuated Salmonella secreting and delivering Brucella ribosomal protein L7/L12 induces humoral and cellular immune responses and protects mice against virulent Brucella abortus 544 challenge
Source: Vet Res. 2020 Jan 23;51:6. doi: 10.1186/s13567-020-0735-y (PMC6979349; doi:10.1186/s13567-020-0735-y)

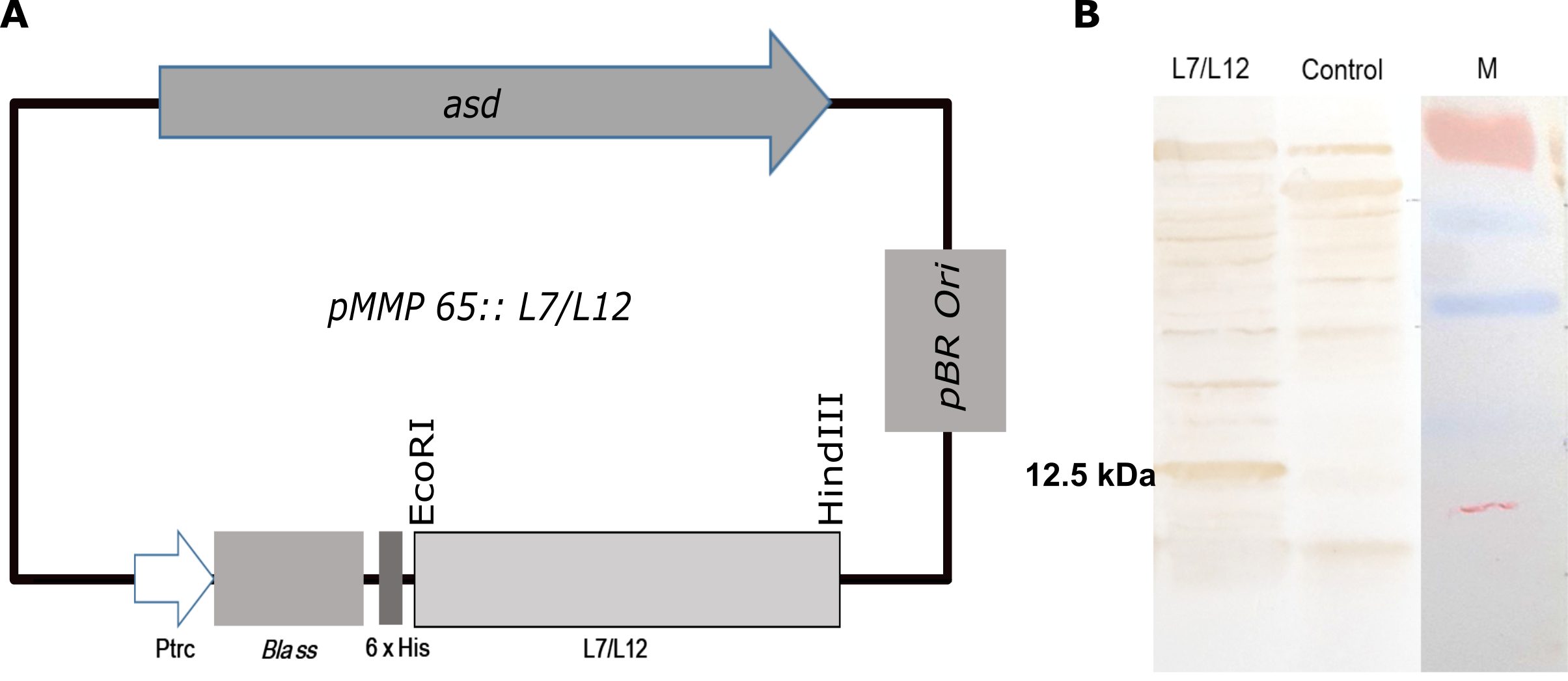

Supplement: Supplementary file 1 — Additional file 1. Graphic representation of plasmid construct and confirmation of protein secretion. (A) Major elements of plasmid pJHL65:: L7/L12 are depicted. Figure is not drawn with actual proportions. (B) Western blot confirmation of protein secretion. Expected size of L7/L12 protein is 12.5 kDa. [file 13567_2020_735_MOESM1_ESM.tif]

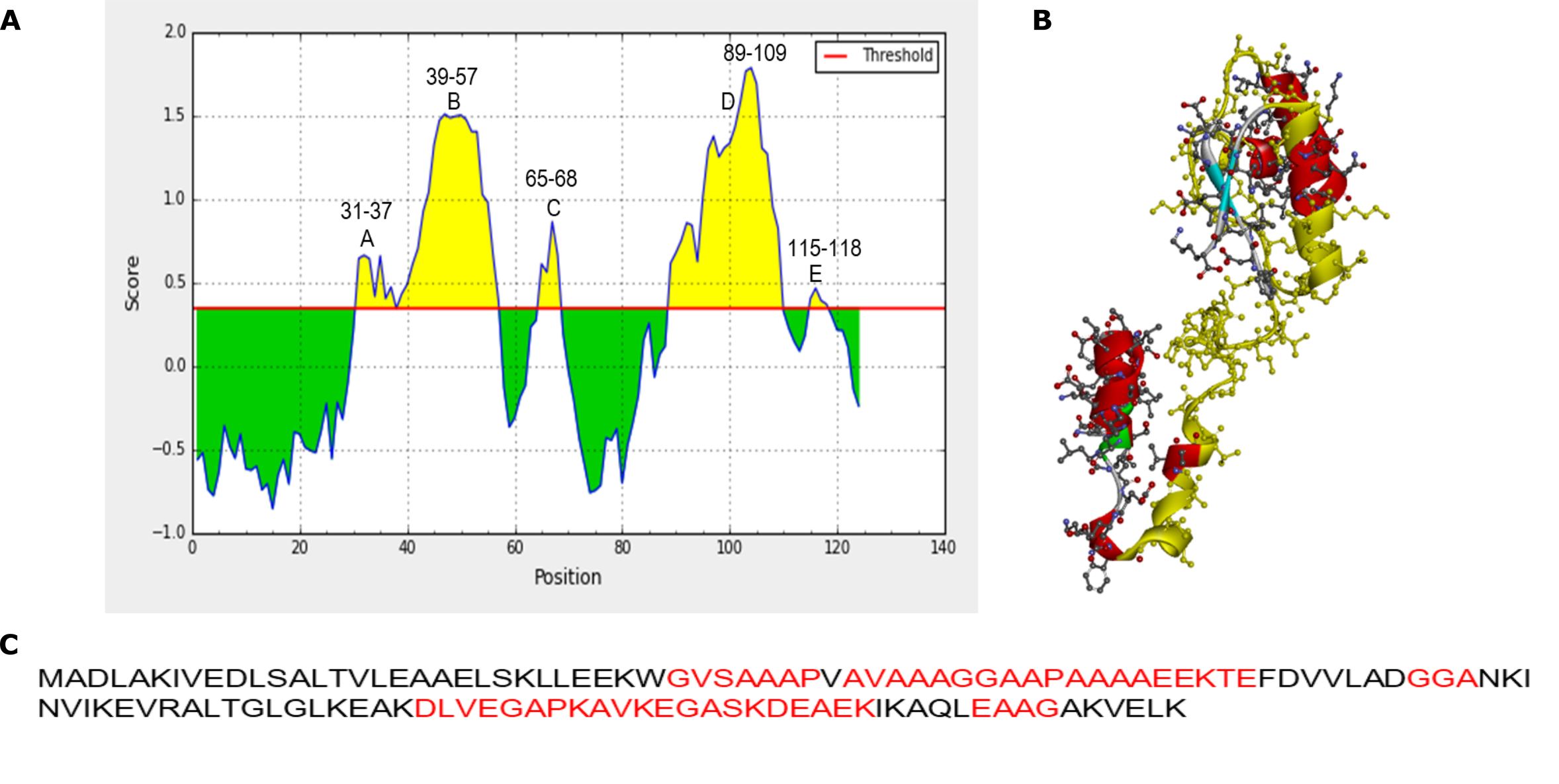

Supplement: Supplementary file 2 — Additional file 2. Epitope analysis of L7/L12 is demonstrated. (A) Graphic representation of predicted epitopes is presented in yellow. (B) Distribution of predicted epitopes on three-dimensional model is demonstrated. (C) Amino acid residues resembled in each epitope are demonstrated in red. Altogether, 5 immunodominant epitopes were predicted. [file 13567_2020_735_MOESM2_ESM.tif]

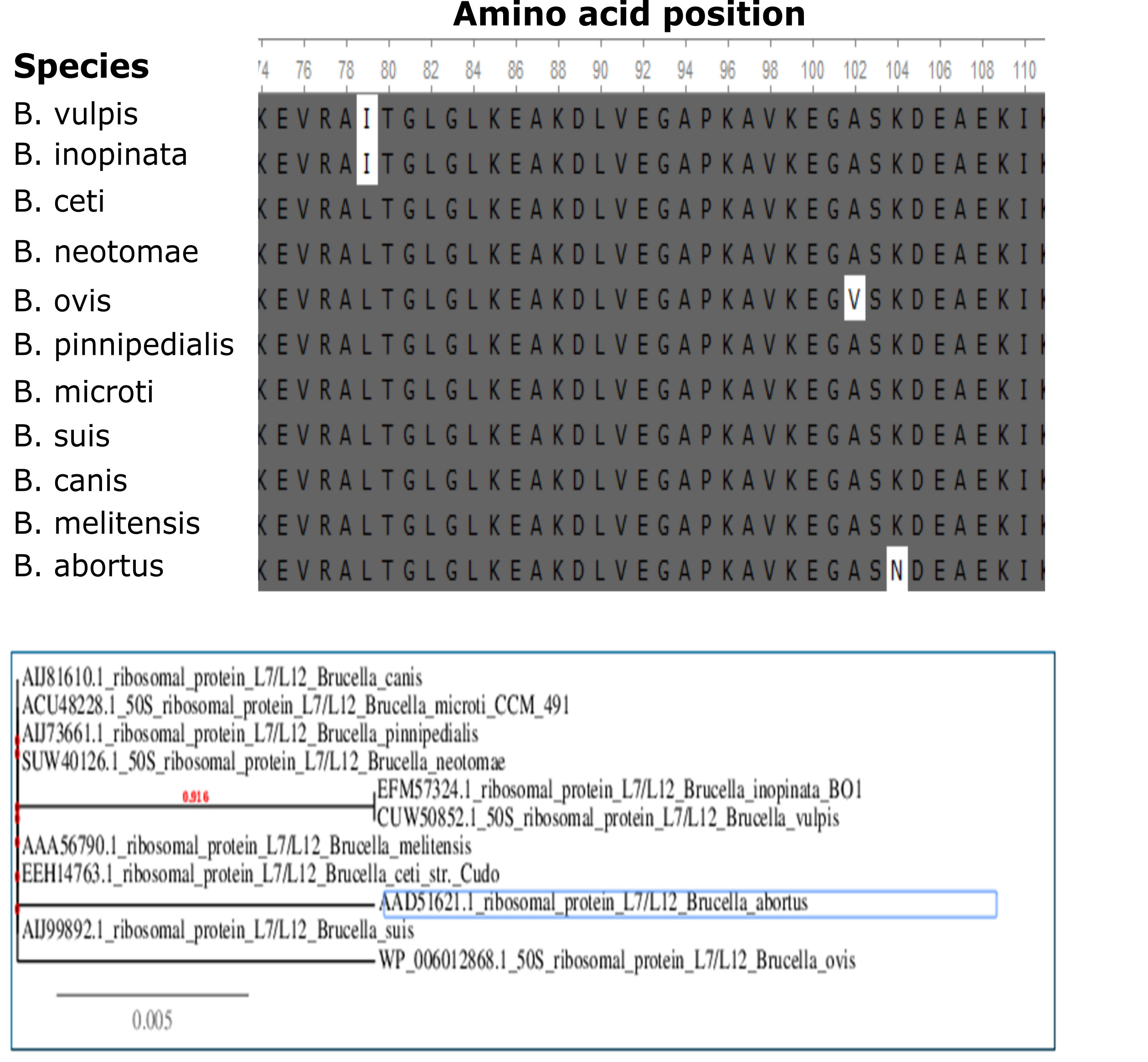

Supplement: Supplementary file 3 — Additional file 3. High degree of L7/L12 sequence conservation is demonstrated within Brucella taxid. Only variable residues were demonstrated in white boxes. Phylogenetic tree constructed on maximum likelihood method also demonstrates extremely high level of sequence identity. Branch length resembles the number of amino acid residue substitutions in each species. Figures were generated by Phylogeny.fr program and Unipro UGENE. [file 13567_2020_735_MOESM3_ESM.tif]

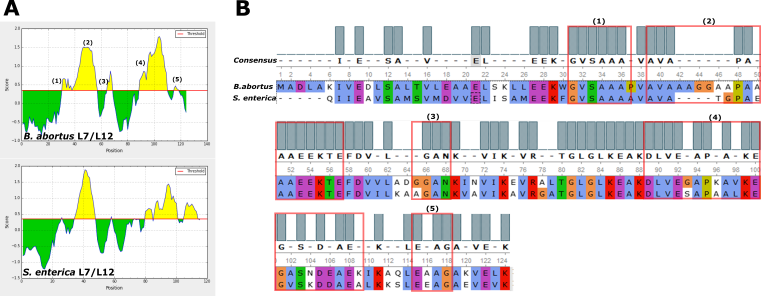

Supplement: Supplementary file 4 — Additional file 4. Sequence homology. (A) The homology of B cell epitopes were compared between Brucella and Salmonella L7/L12 antigen. (B) The sequence identity was determined between Brucella and Salmonella L7/L12 antigen using Muscle algorithm in Ugene software. The squares depict the predicted B-cell epitopes on Brucella L7/L12 antigen. [file 13567_2020_735_MOESM4_ESM.tif]
